# Organic Food Conclusions Don’t Tell the Whole Story

**DOI:** 10.1289/ehp.120-a458

**Published:** 2012-12-03

**Authors:** David C. Holzman

**Affiliations:** David C. Holzman writes on science, medicine, energy, economics, and cars from Lexington and Wellfleet, MA. His work has appeared in *Smithsonian*, *The Atlantic Monthly*, and the *Journal of the National Cancer Institute*.

A widely reported Stanford University study^1^ concluding there is little difference in the healthfulness and safety of conventional and organic foods has been criticized by experts in the environmental health sciences for overlooking the growing body of evidence on the adverse effects of pesticides. Critics take to task the authors’ omission of relevant studies and overinterpretation of the data.

The meta-analysis of 237 studies, published in the September 2012 *Annals of Internal Medicine*, largely focused on nutrient content and viral/bacterial/fungal contamination of organic versus conventionally grown foods. Nine studies reporting pesticide residues, including three of residues exceeding federal limits, were included in summary analyses.

The authors concluded that the studies reviewed do not support what they call the “widespread perception” that organic foods overall are nutritionally superior to conventional ones, although eating an organic diet may reduce exposures to pesticides and antibiotic-resistant bacteria.^1^ A Stanford press release quoted senior author Dena Bravata as saying, “There isn’t much difference between organic and conventional foods, if you’re an adult and making a decision based solely on your health.”^2^ (According to the Stanford Medical Center press office, Bravata is no longer doing interviews about the study.)

In one key finding, the team reported a “risk difference” of 30% between conventional and organic produce, meaning organic produce had a 30% lower risk of pesticide contamination than conventional produce. That number was based on the difference between the percentages of conventional and organic food samples across studies with any detectible pesticide residues (38% and 7%, respectively).

But the concept of risk difference is potentially misleading in this context, as the metric does not refer to health risk, according to Charles Benbrook, research professor and program leader for Measure to Manage (M2M): Farm and Food Diagnostics for Sustainability and Health at Washington State University. Furthermore, says Benbrook, “Pesticide dietary risk is a function of many factors, including the number of residues, their levels, and pesticide toxicity,” not just whether contamination was present.

In a letter accepted for publication in the *Annals of Internal Medicine*,^3^ Benbrook pointed to the Stanford team’s lack of consideration of extensive government data on the number, frequency, potential combinations, and associated health risks of pesticide residues in U.S. food. Using data from the U.S. Department of Agriculture’s Pesticide Data Program,^4^ Benbrook calculated a 94% reduction in health risk attributable to eating organic forms of six pesticide-intensive fruits.^3^

The Stanford researchers also missed opportunities to examine the relationship of pesticides and health outcomes demonstrated in a growing number of cohort studies, says Brenda Eskenazi, a professor in the School of Public Health at the University of California, Berkeley. Eskenazi conducted one such study,^5^ one of a trio published in April 2011 that examined the relationship between cognitive development and prenatal pesticide exposures in two multiethnic inner-city populations^6^^,^^7^ and one farmworker community in California.^5^ One of the studies^7^ found deficits of seven IQ points in 7-year-old children in the highest quintile of pesticide exposure, compared with children in the lowest quintile, as measured by maternal urinary pesticide metabolite levels during pregnancy. Results were comparable in the other two studies.

In concluding that the evidence “does not suggest marked health benefits from consuming organic versus conventional foods,”^1^ many commenters, including Eskenazi and Benbrook, felt the Stanford team ignored risks to broader public health like those outlined in an April 2012 review by David C. Bellinger, a professor of neurology at Harvard Medical School. In his review Bellinger argued that subtle impacts of organophosphate pesticides on neurodevelopment can add up to substantial population-level impacts. He wrote, “It is frequently noted that a modest downward shift in mean IQ scores will be accompanied by a substantial increase in the percentage of individuals with extremely low scores.”^8^

Conventional toxicology testing is now being shown to miss responses that occur at doses that are orders of magnitude lower than previously established no-observed-adverse-effects levels,^9^ with potential implications for our understanding of pesticide safety. And others are finding in animal studies that pesticide exposures *in utero* can induce epigenetic changes that alter stress responses and disease rates in future generations.

In one study, exposure of rats to vinclozolin, a common agricultural fungicide, was associated with altered stress responses in the F3 generation (the original animals’ great grandchildren), compared with F3 progeny of unexposed animals.^10^ These responses were seen at high doses unlikely to be encountered as food residues but potentially applicable to agricultural workers. Exposures to the pesticides methoxychlor, DEET, permethrin, and vinclozolin, as well as dioxin (which can appear as an impurity in pesticides), also “predispose animals to develop a variety of adult-onset diseases earlier than normal,” says Michael Skinner, a professor in the Washington State University School of Biological Sciences who coauthored this study. He says these effects are “still detectable in animals over four subsequent generations, without diminution.”

In October 2012 the American Academy of Pediatrics weighed in, for the first time ever, on the question of whether children benefit from an organic diet.^11^ In a report published in *Pediatrics*, the academy recognized that an organic diet definitely reduces exposure to pesticides and may reduce diseases associated with antibiotic resistance but has not been proven to offer a clinically relevant nutritional advantage over a conventional diet. The academy emphasized the importance of providing children a diet rich in fruits, vegetables, whole grains, and low-fat or fat-free dairy products, regardless of whether the foods are conventional or organic, and provided resources for parents seeking guidance on which foods tend to have the heaviest pesticide residues.
